# Malaria systems immunology: *Plasmodium vivax* induces tolerance during primary infection through dysregulation of neutrophils and dendritic cells

**DOI:** 10.1016/j.jinf.2018.09.005

**Published:** 2018-11

**Authors:** Andres F. Vallejo, Robert C. Read, Myriam Arevalo-Herrera, Sócrates Herrera, Tim Elliott, Marta E. Polak

**Affiliations:** a Clinical and Experimental Sciences and NIHR Southampton Biomedical Research Centre, Faculty of Medicine, University of Southampton, Southampton General Hospital, LE59, MP813, SO16 6YD, Southampton, UK; b Caucaseco Scientific Research Center, Cali, 760043, Colombia; c School of Health, Universidad del Valle, Cali, 76001, Colombia; d Centre for Cancer Immunology, Faculty of Medicine, University of Southampton, SO16 6YD Southampton, UK; e Institute for Life Sciences, University of Southampton, SO17 1BJ, UK

**Keywords:** Malaria, Controlled human malaria infection, Transcriptomics, Dendritic cells, Immunoregulation, Vaccines

## Abstract

**Objectives:**

To dissect the transcriptional networks underpinning immune cells responses during primary *Plasmodium vivax* infection of healthy human adults.

**Methods:**

We conducted network co-expression analysis of next-generation RNA sequencing data from whole blood from *P. vivax* and *P. falciparum* controlled human malaria infection (CHMI) of healthy naïve and malaria-exposed volunteers. Single cell transcription signatures were used to deconvolute the bulk RNA-Seq data into cell-specific signals.

**Results:**

Initial exposure to *P. vivax* induced activation of innate immunity, including efficient antigen presentation and complement activation. However, this effect was accompanied by strong immunosuppression mediated by dendritic cells via the induction of Indoleamine 2,3-Dioxygenase 1(IDO1) and Lymphocyte Activation Gene 3 (LAG3). Additionally, *P. vivax* induced depletion of neutrophil populations associated with down regulation of 3G-protein coupled receptors, CRXCR1, CXCR2 and CSF3R. Accordingly, in malaria-exposed volunteers the inflammatory response was attenuated, with a decreased class II antigen presentation in dendritic cells. While the immunosuppressive signalling was maintained between plasmodium species, response to *P. falciparum* was significantly more immunogenic.

**Conclusions:**

*In silico* analyses suggest that primary infection with *P. vivax* induces potent immunosuppression mediated by dendritic cells, conditioning subsequent anti-malarial immune responses. Targeting immune evasion mechanisms could be an effective alternative for improving vaccine efficacy.

## Highlights

• *In silico* analysis suggests that primary infection with *P. vivax* induces potent immunosuppression mediated by dendritic cells.
• Antigen presentation is attenuated in malaria-experienced vs malaria-naïve individuals
• Malaria induce immunosuppressive effect manifested with strong induction of *IDO1*.

## Introduction

Malaria remains an important public health problem worldwide, with more than 216 million cases per year 445,000 deaths.^1^ The cyclical fever and pro-inflammatory state associated with malaria has been postulated to inhibit establishment and maintenance of immunological memory^2^; indeed, humoral and cellular responses to *Plasmodium* antigens are inefficiently generated and rapidly lost in the absence of ongoing exposure. As a result, individuals from high malaria transmission areas develop partial protection against severe symptoms at an early age and experience a significant number of asymptomatic infections afterwards.^3^ In these infections host immunity can reduce parasitaemia, but parasite clearance is not achieved.

There is evidence that *Plasmodium falciparum,* the most investigated malaria species, can modulate immune responses by interfering with maturation of antigen-presenting cells, however the precise mechanisms leading to immune tolerance are poorly understood. Likewise, it has been suggested that malaria parasites impair dendritic cells (DC) function, resulting in the induction of tolerant T cell phenotype.^4^ DCs can exert an immunosuppressive effect by increased surface expression of immune checkpoint proteins such as Programmed Cell Death 1(PD1) or production of tolerogenic substances such as indoleamine 2,3-dioxygenase (IDO1). It has been shown that DC uptake of infected red blood cells (iRBC) impairs the immune responses during blood stage malaria by interfering with the priming and elicitation of liver-stage immunity.^5^, ^6^ During the chronic phase of the infection, the inhibitory molecule IDO1 is up-regulated in DC, inducing PD1 and LAG-3 expression in CD4 T cells, interfering with the memory acquisition.^7^, ^8^, ^9^

Immunity to *P. vivax* is more rapidly acquired than immunity to *P. falciparum*.^10^ Whereas a single infection with *P. vivax* usually results in a strongly reduced incidence of febrile episodes upon re-infection, secondary *P. falciparum* infections are associated with fever and high parasitaemia. Comparing the immune responses induced by each parasite could reveal immune evasion mechanisms to be used as targets to increase the vaccine efficacy. Unlike *P. falciparum*, there are no established laboratory methods to culture *P. vivax* in vitro^11^ which has retarded study of this parasite. Controlled human malaria infection (CHMI) models allow precise study of host-parasite interactions because they lack the wide biological variation associated with natural disease, such as strain-to-strain variation, host co-morbidities and time to presentation etc.^12^ Systems immunology analysis of CHMI models offers unique opportunities to study human immune responses during malaria infections by identifying major molecular players at each stage, providing a detailed and comprehensive understanding of the complex host-parasite interaction. Previously, we have developed systems immunology pipelines which allowed us to identify the molecular basis of the orchestration of immunity by DC, revealing potential molecular targets for immune interventions.^13^, ^14^ Similar approaches have led to identification of molecular signatures capable of predicting the efficacy of vaccine-induced immunity and examining the transcriptional and cellular responses in *P. falciparum* CHMI models.^15^, ^16^

To better understand immune responses induced by exposure to *P. vivax* we conducted systems immunology analyses of our publicly available RNA-Seq data from a *P. vivax* CHMI in order to elucidate key host-parasite interactions as potential vaccine targets. We optimised bioinformatic pipelines to enhance read-alignment and increase the number of identified transcripts, improving the sensitivity of the analysis. By using cell specific signatures obtained from single cells, we deconvoluted the signal from whole blood to specific cell types. Network analysis and *in silico* signal deconvolution allowed us to identify the role of DC in induced tolerance as well as specific targets in the antigen presentation pathway that could play a central role in establishing of immunological memory. Finally, we compared the *P. vivax* transcription programs with a similar CHMI in *P. falciparum* to define the specificity of the tolerogenic responses.

## Methods

### Systems immunology analysis of RNA-Seq data from malaria CHMI

Our *P. vivax* dataset is deposited in Gene Expression Omnibus (GEO) under accession number GSE67184. The dataset is associated with a *P. vivax* CHMI, corresponding to the analysis of differences in gene expression between malaria-naïve (MN) and malaria-exposed (ME) volunteers. After challenge with *P. vivax*, whole blood transcriptomes from six naïve volunteers, were compared with six malaria-exposed to investigate the greater symptom severity in naïve infection, which occurs despite equivalent parasite loads and time to diagnosis.^17^ The RNA-seq data was derived from 12 whole blood samples taken at two time points (before and 7 days post inoculation of human volunteers with *P. vivax* as described in.^17^ The *P. falciparum* dataset deposited in Gene Expression Omnibus (GEO) under accession number GSE50957 is associated with a *P. falciparum* CHMI, were PBMC transcriptomic profiles were obtained to explore the association of malaria immunity with fever.^18^

### Next Generation Sequencing data processing

All reads were subject to quality control using FastQC.^19^ Sequences were trimmed to remove adapter contamination and low-quality nucleotides using Trimmomatic^20^ and only reads with quality scores  ≥10 and  ≥40 bp length were kept for further analysis. Hisat2^21^ was used to align each sample against a reference genome (GRCh38.p11). The alignment result files (bam files) were sorted and indexed using SAMtools.^22^ Alignments were counted for each gene using the HTSeq count^23^ (**Table S1**). For differential expression analysis and gene co-expression network analysis, gene expression was estimated as counts per million (CPM), filtering out genes lees that two gene counts in at least half of the samples were used. Determination of differentially expressed genes (DEG) was done using *EdgeR*^24^ with a nested paired design. The expected FDR was estimated using the Benjamini-and-Hochberg method. A P adj  ≤0.05 was considered to be significant. TPM (Transcripts per million) was used for comparing gene expression levels.

### Gene co-expression network analysis

Un-supervised transcript co-expression analysis was performed using the graphical correlation-based tool BioLayout *Express*^3D^^25^ to visualize clusters or subgroups of genes that shared similar patterns of expression across different samples. Unlogged expression values pre-filtered for low expressed genes was used as input. A range of correlation coefficients (*r* = 0.8 to *r* = 0.95) and Markov Clustering Algorithm values were used (MCL = 1.7 − MCL = 5) to determine an optimal graph structure. Clusters were then manually curated to remove artefacts. The gene clusters with highest correlation scores were used to generate and visualize networks based on GO-enrichment analysis (GOEA) by using ToppGene.^26^ Weighted gene set enrichment analysis (WGSEA) was performed using GeneTrial^27^ using as input the DEGs from EdgeR. WGSEA was performed using a Kolmogorov–Smirnov non-parametric rank statistic with Benjamini and Yekutieli FDR multiple testing adjustment method. Gene lists were ranked based on fold change (keeping the dysregulation direction) with 1 × 10^6^ permutations. The significance level was set at 0.05. Transcription factors were identified using the transcriptional regulatory relationships deposited in the TRRUST database.^28^ STRING 10.5^29^ was used generating the edges based (score > 0.6) and PPI network building. Cytoscape 3.5 was used to create the network.

### Construction of cell type specific signatures from single cell RNA-Seq information to deconvolute bulk RNA-Seq samples

To evaluate gene-specific differences between blood cells we retrieved the Zheng et al. SC-RNA-Seq dataset.^30^ This dataset contains gene expression profiles of 8K human peripheral blood mononuclear cells (PBMC), annotated based on similarity with 4 immune cell subpopulations (myeloid DC (mDC), B cells, T cells and NK cells). We extracted marker genes for each cell type based on high expression, high variation (Fano factor above mean-dependent threshold), and cell-type restricted (*p* < 10^−5^, defined by a Kolmogorov–Smirnov test). Because of a sample size requirement, we merged the original single cell data from T cell groups (CD4 and CD8) into one group. Neutrophil markers were obtained from a similar analysis using the Hoek *et al.* RNA-Seq dataset.^31^ The cell type-specific markers were applied to obtain a reference basis matrix which was used to infer the composition of bulk tissue samples from their total gene expression using CIBERSORT deconvolution method^32^ with gene expression values without log-transformation as input. The dataset GEO entry (GSE60424) which contains raw RNA-Seq and sample annotation data form 6 immune cell subsets and whole blood was used for validating the signature.

### Statistical analysis

Statistical analyses were performed using Prism 7 (GraphPad Software) and methods embedded in bioinformatic pipelines. The Shapiro–Wilk test was used to test for normality. For non-normally distributed data the Wilcoxon matched-pairs signed rank test was used for comparison of two groups. For normally distributed data a paired student's *t* test was used. The statistical test used and p values are described in the figure legends with *p < 0.05, **p < 0.01, and ***p < 0.001.

## Results

### Transcriptional profile analysis indicates development of innate immune responses during the first *P. vivax* infection in naïve individuals

**Fig. 1:**
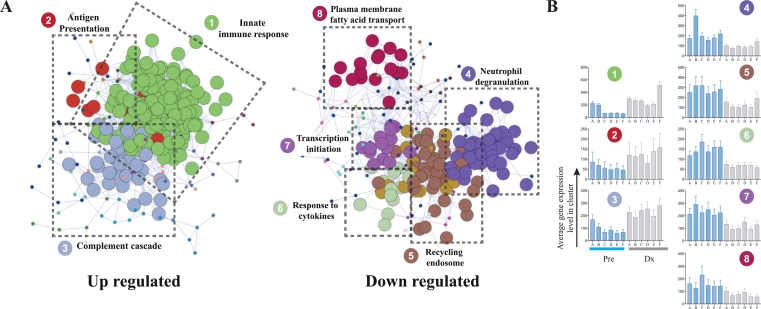
Gene co-expression analysis (CEA) indicates development of immunosuppression during *P. vivax* infection in naïve individuals. (A) Visual representation of whole transcriptome analysis of whole blood from human naïve volunteers during the controlled human malaria infection (CHMI). The analysis identified eight main clusters. (BioLayoutExpress3D, *r* = 0.92, MCL = 1.7). Lines (edges) represent the similarity between transcript expressions; circles (nodes) represent transcripts. Clusters of co-expressed genes are coded by colour. Enrichment of gene ontology terms in clusters was done using ToppGene^26^) Mean (± SEM) expression profiles for clusters 1–8, pre-challenge (pre, blue bars) and on diagnosis day (Dx, grey bar). (B) Mean (±SEM) expression profiles for clusters 1–8, pre challenge (blue bars) and at the diagnosis (grey bars). (For interpretation of the references to colour in this figure, the reader is referred to the web version of this article.)

Our previous analysis of differences in gene expression between malaria-naïve (MN) and malaria-exposed (ME) volunteers demonstrated significant changes in gene expression at the time of malaria diagnosis, particularly in the naïve volunteers, with downregulation of genes related to innate immunity, and inflammation.^12^ We performed paired differential gene expression analysis comparing MN individuals (*n *= 6) before and after the challenge, to establish the transcriptomic networks activated during malaria infection. A total of 1,072 DEGs (EdgeR, FDR *p* < 0.05) were identified, of which 39% were upregulated (**Table S2**). Co-expression analysis (CEA) identified eight main clusters (576 genes, Fig. 1(A)) split into 2 major isolated network structures, comprising up-regulated and down-regulated genes (BioLayout *Express*^3D^, *r* = 0.92, MCL = 1.7). Three largest clusters were made of 202 genes up-regulated on exposure to malaria and involved in innate immune responses (FDR = 9.4 × 10^−33^). Cluster 1, characterised by the highest average gene expression level at diagnosis, recapitulated the typical inflammation pattern including up-regulation of *CXCL9, CXCL10, IFIT1, IFIT2, IFIT3, IFIT5, IRF1* and *IRF7* (Fig. 1(B)). Cluster 2 and 3 contained co-expressed genes involved in antigen presentation, including *HLA-DMB* and *CD74* (Cluster 2) and complement activation (Cluster 3). In contrast, cytokine response and endosome genes were downregulated during the infection (Clusters 4-6). Importantly, Cluster 4 comprised significantly down-regulated neutrophil-associated genes, including the neutrophil chemoattractants *CXCR1, CXCR2* and *CSF3R,* which promotes neutrophil maturation.^33^ Notably, these three G protein-coupled receptors have similar affinities to cytokines and chemokines and share structural conformation with the *ACKR1 (DARC)*, the *P. vivax* receptor in reticulocytes.^34^^,^^35^ Other molecules with immune function that were down-regulated (in cluster 4) included the receptor *CD163*,^36^ involved in haemoglobin clearance; *CR1*, a regulator of the complement cascade, *CCR3* a receptor that binds to CCL11 and *NCF4* involved in activates flavocytochrome b in neutrophils.

### Antigen presentation is attenuated in malaria-experienced vs malaria-naïve individuals

**Fig. 2:**
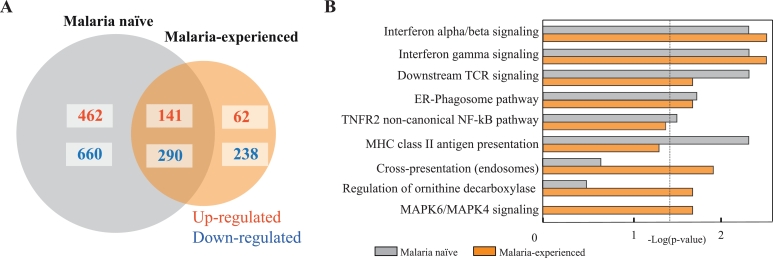
Changes in gene expression during malaria infection in malaria naive and malaria-exposed volunteers. (A) Comparison of differentially expressed genes induced during *P. vivax* infection in naïve vs malaria-experienced volunteers, red: up-regulated genes, blue: down-regulated genes. (DEG identification: EdgeR, FDR *p* value <0.05) (B) Overexpressed biological pathways (REACTOME database) identified using weighted gene-set enrichment analysis (WGSEA) comparing MN and ME individuals using a Kolmogorov–Smirnov non-parametric rank statistic with Benjamini and Yekutieli FDR multiple testing adjustment method (significance level was set at 0.05). Gene lists were ranked based on fold change with 1 × 10^6^ permutations. (For interpretation of the references to colour in this figure, the reader is referred to the web version of this article.)

In order to profile the changes in the immune response in malaria-exposed individuals during a *P. vivax* CHMI, we compared the genes differentially expressed upon *P. vivax* challenge in MN and ME individuals. A total of 400 DEGs were detected in ME individuals (FDR < 0.05), from which 30% were up regulated (Table S3). Importantly, ME volunteers showed 62% fewer DEGs compared with the MN volunteers with a lower proportion of upregulated genes (Fig. 2(A)). To determine biological processes either enriched or depleted due to differential malaria exposure, modules of co-expressed genes associated with ME and MN response patterns identified with WGSEA were correlated with Reactome pathways^37^ as gene sets. The functional responses elicited were similar in both groups: *P. vivax* infection induced strong inflammatory responses including IFN-α/ IFN-γ and TCR pathway activation (Fig. 2(B)). However, notable differences between ME and MN transcriptomic responses were observed in antigen processing and presentation. Importantly, class II antigen presentation signature, strongly up-regulated upon the first encounter with *Plasmodium* in malaria-naïve individuals, was not significantly enriched in ME. In contrast, significant enrichment in cross-presentation and antigen processing, regulation of ornithine decarboxylase, and MAPK6/MAPK4 signalling was observed only in ME individuals.

### Single cell-based deconvolution of bulk gene expression samples confirms depletion of neutrophil populations and a significant expansion of dendritic cells

**Fig. 3:**
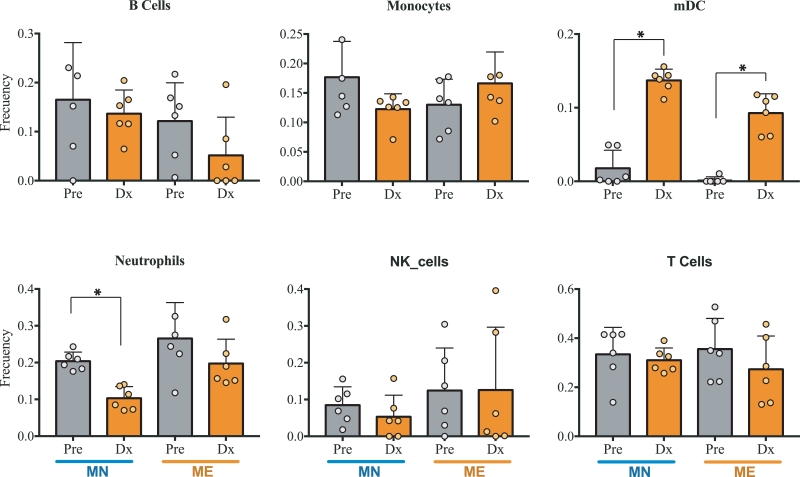
Bulk RNA sample deconvolution into specific cell proportions. Estimated proportions of B cells, monocytes, mDC, neutrophils, NK and T cells based on single-cell-specific gene expression (*n* = 5, unpaired *t*-test; *P < 0.05) in naïve (MN) and malaria-exposed (ME) pre (grey bars) and post (orange bars) exposure to *P vivax*. Mean of *n* = 6  ±  SEM shown. (For interpretation of the references to colour in this figure, the reader is referred to the web version of this article.)

The availability of single-cell gene expression data offers improved means for deconvolution by providing profiles from a large number of minimally perturbed primary cells. To estimate the proportions of B cells, monocytes, mDC, neutrophils, NK and T cells in the whole blood samples obtained during the CHMI trial, we optimized and validated a bioinformatics pipeline for dissecting the characteristic expression profiles of immune cell types using a data set comprising 8K single cells from a healthy donor.^30^ Based on this analysis we built a deconvolution matrix containing 443 specific markers for 5 cell types (Table S4). Information on specific cell-type transcriptomic expression is captured by the single cell deconvolution matrix (SC-matrix) as shown by the (Fig. S1). We used the SC-matrix to assess the relative proportions of immune cells during malaria infection of naïve individuals and individuals with prior exposure. While estimated frequency of B cells, T cells, monocytes, and NK cells remained stable between naïve and previously-exposed individuals, pre and post challenge, proportion of DC signal dramatically increased upon challenge with *P. vivax* in both groups (Fig. 3). This indicates, that the biology of blood DCs is uniquely regulated during malaria infection, and identifies the cellular source of the changes in antigen processing and presentation signal, detected in co-expression analysis (Fig. 1). Furthermore, we observed a significant decrease in the neutrophil population in MN at the time of malaria diagnosis, (Fig. 3). This decrease in neutrophils is consistent with the significant downregulation of *CXCR1, CXCR2* and *CSF3R* (Fig. S3). A smaller, statistically insignificant, decrease was observed for the ME group. Both groups exhibited a significant increase in expression of *CXCL9* and *CXCL10* during the infection which could be correlated with the levels in T cell and monocytes. The receptor of the monocyte chemoattractant protein-1, *CCR2*, was up-regulated only in MN individuals which correlates with the high predicted levels at the time of diagnosis (Tables S2 and 3).

### P. *vivax*-induced immunosuppression is associated with IDO1 expression and decrease of class II antigen presentation

**Fig. 4:**
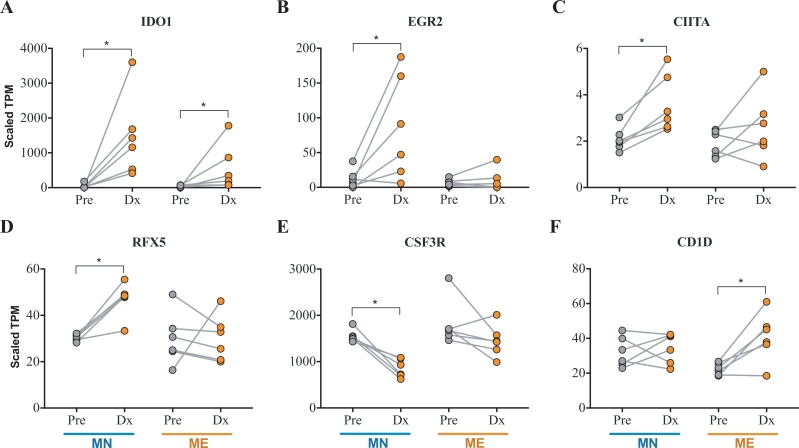
*P. vivax* induced immunosuppression is mediated by IDO1. Paired analysis of key regulatory genes during a *P. vivax* CHMI (*n* = 5, paired *t*-test; *P < 0.05). Normalised gene counts (scaled TPM) shown in naïve (MN) and malaria-exposed (ME) Pre (grey dots) and post (orange dots) exposure to *P vivax*. (For interpretation of the references to colour in this figure, the reader is referred to the web version of this article.)

An essential function of DCs is to prime naïve T cells, shaping the immune response to be tolerogenic or inflammatory depending on the DCs’ activation phenotype.^38^ While initial exposure to malaria resulted in strong innate immune system activation (Fig. 1) and expansion of blood DC signal (Fig. 3), strong immunosuppressive signaling was observed in parallel. The immunosuppressive effect induced by the first malaria infection was manifested with strong induction of *IDO1* (the top up-regulated transcript, FC = 55.3, Padj = 7 × 10^−7^), correlating with the expression of *EGR2,* a transcription factor binding to *IDO1* promoter (Fig. 4(A) and (B)). IDO1 has been shown to be mainly expressed in DC^39^ and is known to induce a tolerogenic signal through the activation of T-cells (Treg), maintaining the homoeostatic balance and controlling immunopathogenesis through the modulation of the excessive inflammatory response.^40^ Lymphocyte-activation gene 3 (LAG3) expressed exclusively in activated T and NK lymphocytes, was upregulated only in MN, is a CD4 homologue that binds MHC class II molecules with very high affinity and has a negative regulatory effect on T cell function and DC maturation (Fig. 4(C)).^41^, ^42^ Analysis of the expression of transcription factors in MN indicated that *IRF1, STAT1* and *STAT2* were the most highly expressed transcription factors activated in similar proportions in both groups, whereas, *E2F1, PLM* and *KLF5* were the transcription factors changing the most after infection. The interferon-induced proteins *IRF1* and *IRF9* were the most up-regulated genes of the interferon-induced family (Fig. S4). Two of the main transcription factors involved in the antigen presentation gene regulatory network, *CIITA* and *RFX5*, where significantly up regulated in MN but not in ME suggesting a less activated antigen presentation in the ME individuals (Fig. 4(D)-(E)). The Colony Stimulating Factor 3 Receptor (*CSF3R*) a receptor that that controls the production, differentiation, and function of granulocytes was significantly down-regulated in MN but not in the ME group (Fig. 4(F)) which correlates with strong drop in the estimated neutrophil abundance.

### Distinctive gene expression profile in *P. falciparum* infections

In order to assess the specificity of the tolerogenic responses detected in *P. vivax* we used the NGS data from Tran et al.,^18^ documenting the gene expression profile in naïve individuals exposed to *P. falciparum* CHMI (*n* = 5). While molecules associated with immunosuppression (*IDO, CD274*) were observed in both datasets, *P. falciparum* induced stronger inflammatory responses with expression of a wide spectrum of chemokines, chemokine receptors and Toll-like receptors, in contrast to *P. vivax*. Toll like receptor 7 was upregulated during the infection with both parasites, whereas *TLR 1-5* and 8 where exclusively upregulated in *P. falciparum. CXCL9* and 10 where highly upregulated in both parasites, whereas *CXCL11* and CCL2, two potent chemoattractant molecules for lymphocytes and monocytes respectively were only upregulated in *P. falciparum* infections.

**Fig. 5:**
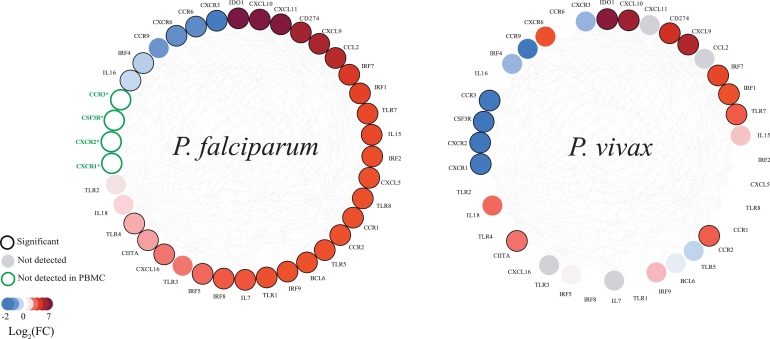
Distinct gene expression profiles in induced by different Plasmodium species Comparative analysis of selected immunomodulatory genes DEG during *P. vivax* and *P. falciparum* CHMI. Mean/median fold change in gene expression level between post and pre-exposure to *P. falciparum* and *P. vivax* shown for each gene. Genes down-regulated on exposure: blue, genes up-regulated on exposure: red. Black outline: significant FDR corrected p value. Green outline: neutrophil-associated genes, not detected in PBMC. Genes are ranked by fold change in *P. falciparum* CHMI. (For interpretation of the references to colour in this figure, the reader is referred to the web version of this article.)

*CXCR3* downregulation on *P. falciparum* could be related with a negative feedback with the *CXCL11. IL15* was the most highly cytokine in *P. falciparum,* whereas in *P. vivax* was *IL18*. While expression of transcription factors involved in activating innate immune responses, including interferon regulatory factors (IRF) 1,2,5,7 and 8 was highly significant in *P. falciparum*, only IRF 1,7 and 9 were significantly up-regulated upon exposure to *P. vivax*. Down-regulation of CXCR1, CXCR2, CCR3 and *CXCL6*, specific to neutrophils, was absent in the *P. falciparum* dataset due to the sample preparation (Fig. 5).

## Discussion

CHMI system is a well-controlled system allowing investigations of immune responses to infection with malaria parasites as they develop in a human host, offering the opportunity to capture the initial programming of systemic immune responses. Applying novel systems immunology approaches to the whole transcriptome data from a unique CHMI study has allowed us, for the first time, to describe molecular and cellular mechanisms potentially involved in malaria-induced immunosuppression.

We have shown that *P. vivax* induces a strong immunosuppression mediated by DCs associated with the induction of *IDO1*. DCs are the key immune regulators keeping in check excessive inflammation to control the cellular immune response through direct contact with effector immune cells and by the production of regulatory cytokines including IL-10 and TGF-β.^43^, ^44^, ^45^ Here, signal deconvolution identified DCs as the cell population most significantly activated by the initial *P. vivax* infection. However, despite the DC potential to prime effective adaptive immune responses, in the context of malaria infection DC induction was associated with immunosuppressive signalling. Thanks to their pivotal role in regulation of immunity, DC could be key for understanding the tolerance induced during the first *P. vivax* malaria infection and could underlie the relatively benign disease experienced in ME individuals. IDO1 has been identified as an important immunoregulator inhibiting T-cell responses and promoting immune tolerance.^46^ In leishmaniasis^47^ and malaria,^48^ IDO mediated attenuation of adaptive immunity may facilitate parasite persistence and disease severity. The high expression levels of *IDO1* are likely to be produced by DCs and in combination with the observed reduction in the HLA class II antigen presentation activity, may be responsible for induction of immunosuppressive responses. Remarkably, studies of *P. vivax* asymptomatic individuals found substantially lower levels of inflammatory and regulatory cytokines,^49^ contrasting with higher levels of the regulatory cytokine interleukin (IL)-10 in Brazilian *P. vivax* malaria patients.^50^ Our data are consistent with previous findings of increased Treg activation in adults with clinical illness and controlled infections of *P. vivax* malaria.^51^, ^52^ Transcriptomic programming of *P. vivax* can be understood better when contrasted with highly immunogenic *P. falciparum* infection. While *P. falciparum* induces both tolerogenic and immunogenic transcriptomic responses, exposure to *P. vivax* seems to be distinctively tolerogenic. This important difference may be explained by differential expression of transcription factors regulating innate immunity. While *P. vivax* induces only two members of IRF family, IRF1 and IRF7, potentially limiting the extend of antigen presentation and cytokine/chemokine production, exposure to *P. falciparum* promotes also IRF5, IRF9 and most importantly IRF8, proven to regulate DC survival and pro-inflammatory function.^53^, ^54^, ^55^ In concordance, *P. falciparum* infection induces broader cytokine, chemokine and TLR spectrum. Importantly, IL-7 and CXCR6, two key molecules involved in memory responses where dysregulated significantly only in *P. falciparum* infections, likely potentiating aberrant immune responses, including survival of memory CD4 + T cells (IL7) and inefficient CD8 memory responses (CXCR6).^56^ This strong inflammatory response induced by *P. falciparum* appears to be essential to expeditiously control a highly pathogenic infection at the expense of the long-term immunity.

Conversely, initial exposure to *P. vivax* induces dramatic drops in neutrophil population, specifically downregulating of *CXCR1, CXCR2* and *CCR3*. Neutrophils regulate DC function during microbial infection, probably by cross-talk between these cell populations as an important component of the innate immune response to infection.^57^ *IL-8 (CXCL8)*, the main ligand of *CXCR1*, is a powerful neutrophil chemotactic factor and its binding to CXCR1 induces activation and migration of neutrophils.^58^, ^59^ Taken together, this data, and the recent finding of *P. vivax* affecting bone marrow^60^ we hypothesise that the parasite infection could have deep effects on haematopoietic progenitor cells expressing structural related G coupled receptors. Elevated levels of *EZH2* at CIITApIV and the resulting increases in CIITApIV H3K27me3 occur in the presence of IFN-γ and leave the proximal promoter inaccessible for transcription factor binding or transcription initiation.^61^ Decreased expression of E2F1 could result in the modulation of EZH2 which results in expression of *CIITA* and MHC II. This observation correlates with the low antibody response observed in sera from the same volunteers tested against a protein microarray comprising ∼50% of the *P. vivax* proteome.^62^ Based in the activation of *EZH2, HDAC7* and CREBBP is possible that the changes induced by the infection could be genomically imprinted. Indeed, case-control and longitudinal studies indicate that children undergoing malaria episodes have increased susceptibility to infection with non-typhoidal Salmonella species and other bacteria.^63^ Plasmodium-infected mice are highly susceptible to infection with non-typhoidal Salmonella species. It has been reported that malaria-induced immune-modulatory haem oxygenase 1 and IL-10 dampen the effector functions of neutrophils and macrophages.^64^, ^65^ Similarly, impaired neutrophil function has been described both in *P. falciparum* and *P. vivax* malaria.^66^

Interestingly, our approach revealed transcriptional networks of gene modules related to type I interferon, innate immunity and T cell signalling. Importantly, these gene networks were associated with specific phenotypes and predicted changes in immune cell populations. Among these pathways, *IDO1* has been shown to modulate the T in response to the parasitic infection.^46^ This suggests that transcriptional programs associated with particular immunological processes might determine the clinical outcome of *P. vivax* malaria. While development of immunological memory facilitates an immediate recall of effector cells which rapidly clear the parasite preventing an excessive inflammatory response and tissue damage, previous studies have shown that release of pro-inflammatory mediators like TNF-a and IFN-γ during malaria infections, potentially can contribute to organ damage.^67^ Thus, the observed induction of the immunosuppression may be an effect of evolutionary adaptation to prevent excessive damaging inflammation. Taken together, these results indicate that the immune response to *P. vivax* infection is tightly associated with previous host malaria experience. This might explain the lack of correlation between protection achieved in CHMI and vaccine efficacy in the field.^68^, ^69^

Applying a combination of whole transcriptome network analysis and cell signature deconvolution allowed us to gain novel insights into the mechanisms underpinning *P. vivax* infection. The most significant limitation in our study is that the deconvolution method is highly dependent on the fidelity of reference profiles which can potentially over- or under-represent the cell types. However, the use of SC-RNA-Seq data as well as the validation of the signature with data sets from purified cell population mitigate this issue, and allow identification of plausible cell-specific immune mechanisms. A better understanding of immune systems in individuals with varying degrees of immunity to *P. vivax* will be useful to improve rational vaccine design and development novel therapeutic interventions. The mechanisms of immunosuppression that we have shown here could be harnessed to improve current malaria vaccines by targeting specific molecules such as the IDO1, to overcome parasite immune evasion.

## Funding

MEP is funded by Sir Henry Dale Fellowship, Wellcome Trust. Grant no 109377/Z/15/Z . AV is funded by the Royal Society grant no CH160056.

## Conflict of interest

The authors declare to have no conflict of interest.

## Appendix Supplementary materials

Image, application 1

Image, application 2

Image, application 3

Image, application 4

Image, application 5
