# Supplementary material for: Malaria systems immunology: Plasmodium vivax induces tolerance during primary infection through dysregulation of neutrophils and dendritic cells
Source: J Infect. 2018 Nov;77(5):440–7. doi: 10.1016/j.jinf.2018.09.005 (PMC6203889; doi:10.1016/j.jinf.2018.09.005)
Supplement: Supplementary file 1 [file mmc1.docx]

**Supplementary figures**

**Figure S1 Single cell deconvolution matrix.** Specific cell-type transcriptomic expression of 5 cell types from a dataset comprising 8K single cells from a healthy donor (30). Marker genes were obtained based on high expression, high variation (Fano factor above mean-dependent threshold), and cell-type restricted (p < 10^−5^, defined by a Kolmogorov-Smirnov test). Neutrophil markers were obtained from a similar analysis (31).


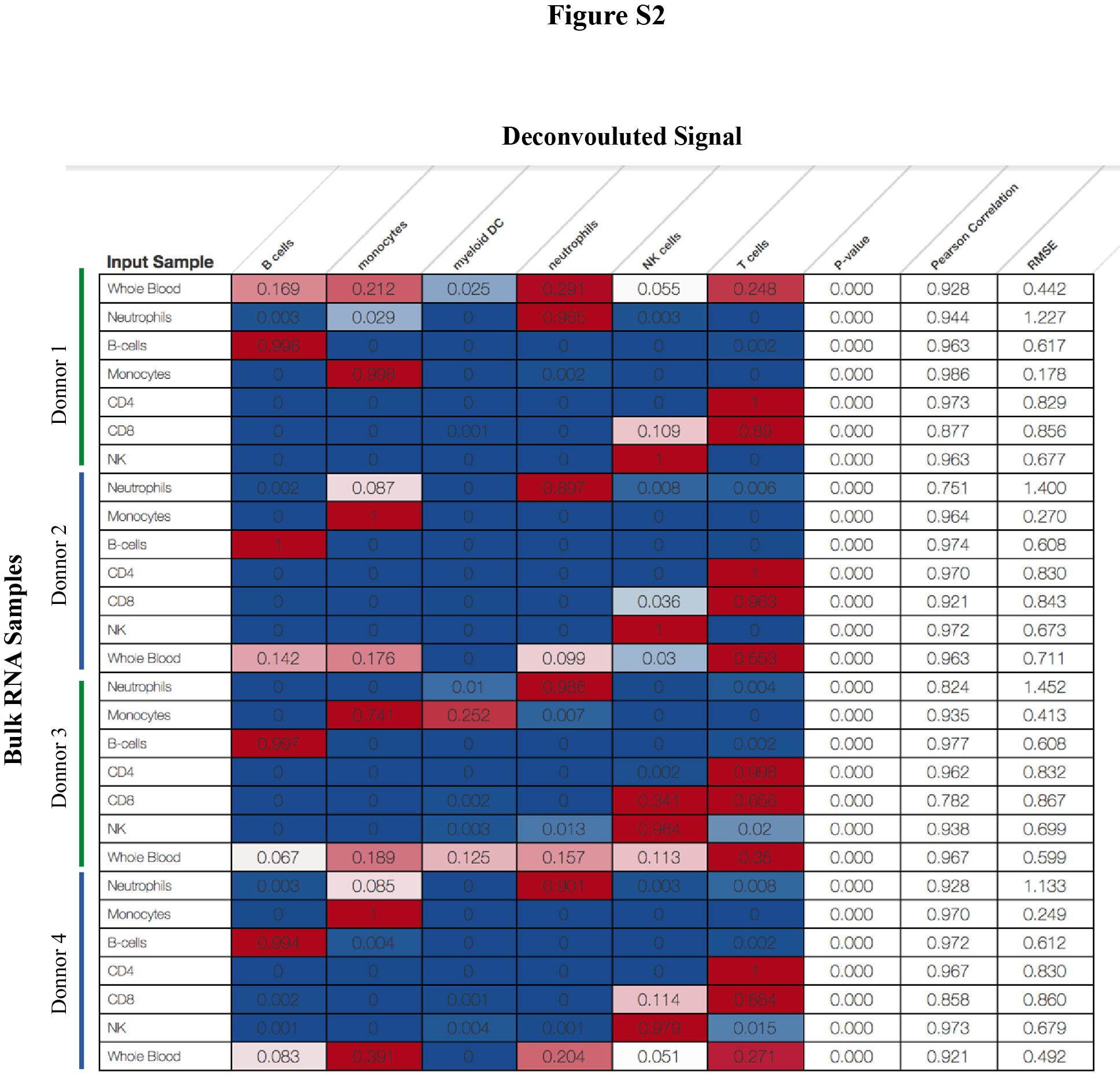


**Figure S2 Validation of the cell specific signature**. A previously published bulk RNA-Seq dataset of five purified immune cells (GSE60424) was analysed using the single cell deconvolution matrix. Estimation of the abundance of member cell types was performed using CIBERSORT (32) with the SC-matrix as signature file.


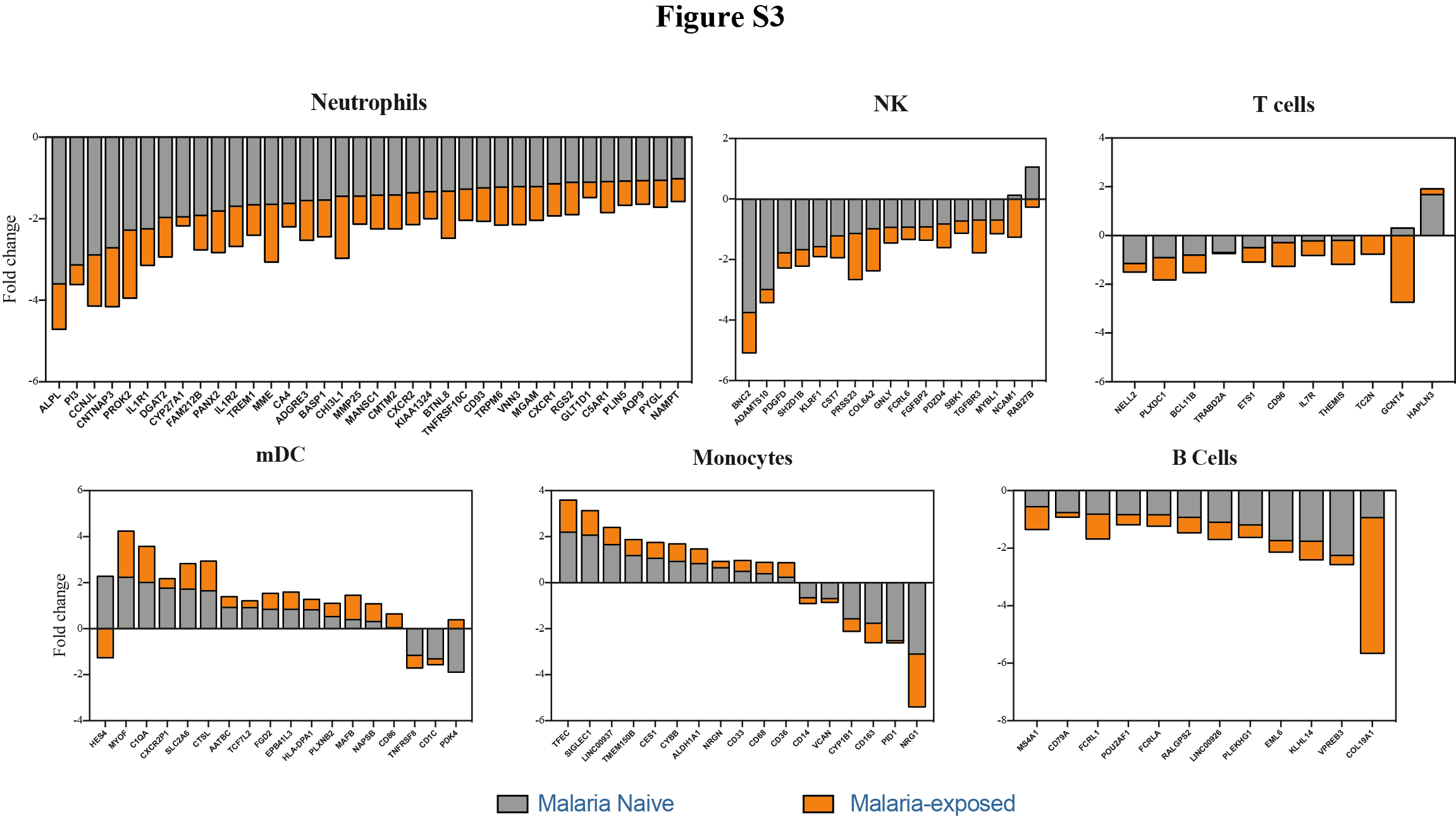


**Figure S3 Cell type specific DEGs**. Using BSEQSC (70) and linear models we estimate the interaction terms between cell proportions and DEGs as the likelihood a particular gene would be expressed in each cell types. Only genes with an FDR p <0.1 were shown.

**Figure S4 Transcription factor network.** A transcription factor network was built by predicted the key transcription factors activated using the TRRUST (28) database, visualized using Cytoscape applying attribute circle layout by fold change. Bars represent the fold changes in Naïve (blue) and malaria exposed volunteers(yellow).

**Reference**

70. Baron M, Veres A, Wolock SL, Faust AL, Gaujoux R, Vetere A, et al. A single-cell transcriptomic map of the human and mouse pancreas reveals inter-and intra-cell population structure. *Cell Syst* 2016; **3** (4):346–60 e4.
